# A phenomenological study of the experiences of nurses working in integrated nursing care wards in Korea

**DOI:** 10.1186/s12912-024-01798-z

**Published:** 2024-02-22

**Authors:** Young-mi Cho, Sun-hui Kim

**Affiliations:** 1 Nursing Science, SunMoon University, 70 Sunmoon-ro 221beon-gil, Tangjeong-myeon, Asan- si, Chungcheonnam-do Korea; 2 Chodang University, 380 Muanro, 58530 Muaneup, Muangun, Jeollanamdo Korea

**Keywords:** Integrated nursing care, Phenomenological approach, Lived experiences, Occupational stress

## Abstract

**Background:**

This study aimed to understand the experiences of nurses working in the integrated nursing care service, a relatively recent addition to the Korean hospital infrastructure, to suggest ways in which to address their grievances and needs and improve their job satisfaction, thereby reducing turnover.

**Methods:**

This study adopted a qualitative approach to explore subjects’ vivid experiences. Data were collected through in-depth interviews with 17 nurses with over one year of experience working in integrated nursing care wards. The main question asked was “Can you describe your experiences in the integrated nursing care ward?” All interviews were recorded, transcribed, and analyzed using Colaizzi’s method for phenomenological research.

**Results:**

Six theme clusters were derived from the analysis: “distorted perceptions of the integrated nursing care ward,” “challenges owing to distorted perceptions of the integrated nursing care ward,” “loneliness and fighting alone,” “being ridiculed,” “practicing textbook holistic care,” and “the satisfaction felt only in the integrated nursing care ward.” For the overarching theme, we identified “Satisfaction in providing holistic care despite the challenges.”

**Conclusions:**

While working in the integrated nursing care ward, nurses practiced holistic nursing care, which in turn built their self-esteem. However, they experienced greater levels of stress as a result of misinformation. Therefore, dissemination of accurate information is necessary to correct public misunderstandings of the integrated nursing care wards. Further, adequate compensation and support systems are needed to relieve the stress nurses felt because of such misunderstandings. Additionally, nurses should be motivated to continue to provide quality care for the patients and take pride in their work. Future research should explore the physical and mental concerns of nurses working in integrated care wards.

## Background

The integrated nursing care service in Korea was established in 2016 by supplementing and modifying a pilot project involving the delivery of comprehensive nursing services to improve the ward environment while providing specialized nursing care [1]. This service aims to reduce the economic burden on patients by eliminating the need for an accompanying family member during hospital admissions while providing them with professional, high-quality care.

Although many countries do not provide integrated nursing care services, most developed countries operate wards without an attendant [2]. In the United States, the attending caregiver does not remain with the patient throughout the course of admission and cannot be in the hospital room after visiting hours. The same practice is followed in Korea’s integrated nursing ward, which involves whole-person care. Moreover, nurse-patient ratios in the other countries are fixed for the provision of high-quality care. In the United States, the ratio of nurses to patients in medical-surgical units is 1:5; it is stipulated that cardiac telemetry nurses cannot care for more than four patients at a time [3]. These nurse-patient ratios differ greatly from the reality in Korea [4, 5]. With the introduction of integrated nursing care services, there has been a steady growth of expectations and concerns voiced in the nursing field. According to the government’s propaganda in the media, integrated nursing care services can reduce patients’ economic burden [6, 7]. In Korea, care for hospitalized patients is provided by family members. Modern Korean families are becoming nuclear, with many dual-income couples, making it difficult for a family member to stay with the patient [8, 9]. Therefore, for inpatients, especially older persons, caregivers are hired [10]. Although caregivers possess a certain amount of education, it is not in professional nursing or medical care, posing a risk of infection and other concerns for patient safety [11–14]. These problems can be resolved if professional nurses perform these additional duties, thereby better securing patient safety.

Several studies have examined burnout and work satisfaction among nurses in general wards [15–17], whose most common stressors were reported to be psychological pain resulting in depression, insomnia, and rapid turnover or retirement of peers [18]. However, no study has considered the unique experiences and characteristics of nurses in the integrated service ward. Previous research on integrated nursing care services focused on the satisfaction and reuse intention of patients [17–19]. Therefore, this study conducted in-depth interviews with nurses working in the integrated nursing care ward to explore their crucial experiences in detail to collect basic data for developing measures to assess the skill level of nurses in the integrated nursing care wards and the systems they adopt to improve the quality of care.

## Methods

### Study design

This qualitative study was designed to discover the essence and meaning behind the phenomenon under study. It applies a phenomenological approach to understand in depth the experiences of nurses working and providing care in the integrated nursing care ward.

To protect the study participants, this research was conducted with the approval of the Bioethics Review Committee of the National Medical Center Institutional Review Board [https://www.nmc.or.kr/instlm/main/contents.do?menuNo=800002] (IRB No. H-1807-092-012). All methods were performed in accordance Bioethics Review Committee of the National Medical Center Institutional Review Board with the recommended guidelines and regulations. Prior to data collection, the purpose and method of the study were explained to the participants in detail, and they were informed that they could withdraw at any point in the study. They were notified that the interview contents would be processed anonymously to maintain confidentiality, and that all identifying information would be coded to ensure anonymity.

### Participants

Participants in this study were recruited using a snowball sampling method, and written informed consent was obtained before interviews were conducted. Participation was restricted to nurses who had worked in the integrated ward for more than a year. Upon completion of the interview, a token of appreciation (a mug) was provided. A total of 17 nurses were recruited before data saturation was achieved (Table 1).

### Collection of data

Data were collected from September to December 2019. All participants were interviewed once, and two were interviewed a second time based on need. Additional information was collected through phone calls. The duration of each interview was between 1 and 2 h. The initial question asked was, “Can you tell us about your experience of working in the integrated nursing care ward?”; a semi-structured interview followed, wherein various questions were added according to each individual’s account. Researchers used their discretion to identify areas for which in-depth information was required.

The interviews were recorded with the participants’ consent, and the contents were transcribed by the researcher. The meaning conveyed by the participants’ words was interpreted by performing multiple readings. Each transcript was directly analyzed, the themes extracted and reviewed with colleagues, and newly emerging questions added to the following interview.

### Data analysis

The data were analyzed according to the phenomenological approach suggested by Colaizzi [20].

1) Description: The recorded contents were transcribed and listened to repeatedly to understand the overall meaning of the experiences of nurses working in the integrated nursing care ward. During the transcription process, the overall meaning of the participants’ experiences was extracted.

2) Coding: Significant statements representing the experiences of nurses in the integrated nursing care ward were selected, yielding 98 main statements.

3) Ordering: The extracted main statements were paraphrased and their interpreted meanings shared with the participants to obtain their confirmation of the essential meaning behind their experience.

4) Theme Extraction: Themes were selected by integrating and categorizing similar derived meanings, and theme clusters were organized by weaving together related themes.

5) Cluster Creation: From the derived meaning, 18 themes and 6 theme clusters emerged.

6) Analysis: The meanings of the clusters and themes were examined and interpreted. Insights relevant to the research topic were extracted.

7) Validation and Structural Description: The nurses’ experiences were integrated, and the participants were asked to check the transcribed content to reconfirm the meaning.

### Ensuring rigor in the research

Appropriate evaluation criteria for qualitative research were used to ensure rigor [18].

First, to increase value, participants with rich experience in the phenomenon were selected and encouraged to express their experiences effectively and thoroughly. Additionally, the transcripts of the interview and the analysis results were shown to the participants for them to check whether their experiences were adequately reflected. The topic derivation process and results were explained to two nursing professors with rich experience in qualitative research, who verified the reliability.

Second, to increase transferability, demographic characteristics of the study participants were collected. Data collection continued until saturation, when no new statements or themes emerged.

Third, to ensure consistency, the process suggested by Colaizzi [20] was discussed in detail, and a final procedure suitable for our study was developed through discussion with a professor familiar with qualitative research.

Fourth, to maintain neutrality, we attempted to control for distortion of the participants’ experiences by receiving continuous feedback from them to eliminate researcher bias and prevent subjective interpretations that were incongruent with the participants’ meaning.

## Results

**Table 1:** Demographic and Characteristics of the Participants (*N* = 17)

Participant ID	Gender	Age (years)	Religion	Marital status	Educational qualification	Total work experience (years)	Current department experience (years)
1	F	34	No	Single	Associate	10.9	2.5
2	F	29	No	Single	Bachelor	5.3	2.3
3	F	27	No	Single	Bachelor	3.8	2.5
4	F	26	No	Single	Bachelor	1.6	1.6
5	F	32	No	Single	Master	7.6	2.6
6	F	46	Yes	Married	Master	22.3	1.7
7	F	27	Yes	Single	Bachelor	2.5	1.8
8	F	31	No	Married	Bachelor	6.7	1.1
9	F	28	No	Single	Bachelor	3.7	2.3
10	F	40	Yes	Married	Master	16.1	1.3
11	F	27	No	Single	Bachelor	3.0	2.2
12	F	31	Yes	Single	Master	4.5	2.0
13	F	29	No	Single	Master	7.3	2.1
14	F	35	Yes	Married	Doctor	11.0	2.0
15	F	29	No	Single	Master	6.2	2.2
16	F	28	No	Married	Bachelor	5.0	1.5
17	F	28	No	Single	Bachelor	4.7	1.8

This study was conducted with 17 nurses working in integrated nursing care wards. All the participants were women, and their mean age was 31.0 ± 5.2 years. Their mean total clinical work experience was 7.19 ± 5.32 years and mean experience in the integrated nursing care ward was 1.97 ± 0.43 years (Table 1).

Analysis of the interview data yielded a total of 18 themes, from which 6 theme clusters with more abstract and inclusive meanings were derived: “distorted perceptions of the integrated nursing care ward,” “challenges owing to distorted perception of the integrated nursing care ward,” “loneliness and fighting alone,” “being ridiculed,” “practicing textbook holistic care,” and “the pride felt only in the integrated nursing care ward” (Table 2).

**Table 2:** Theme Clusters and Themes of the Nurses’ Experience in the Integrated Nursing Ward

Theme Cluster	Theme
Distorted perceptions of the integrated nursing care ward	Being treated like an attendant
	High expectations from family and patients
	Patients who do not hesitate to ask for personal favors
Challenges owing to distorted perceptions of the integrated nursing care ward	Being confused about roles and identity
	Feeling the urge to resign
	Experiencing physical symptoms of stress
Loneliness and fighting alone	Unable to leave the ward even for a moment due to the fear of patients falling
	Responsibility rests solely with the nurse
Humiliation and harassment by patients	Exposure to verbal abuse and sexual harassment
	Patients’ different behaviors to their face and behind their back
	Being ridiculed
Practicing textbook holistic care	Assessing the patient from head to toe
	Caring for patients attentively and specifically
	Quickly detecting the patient’s condition
The pride felt only in the integrated nursing care ward	High patient satisfaction
	Gratitude for reducing the burden on the family
	Building trust

### Theme cluster 1: distorted perceptions of the integrated nursing care ward

This theme cluster represents the nurses’ experiences of ill treatment due to the distorted perceptions of others of their role in the integrated nursing care wards. Patients and their families sometimes have the unrealistic expectation that their fee entitles them to have everything done for the patient. Owing to this misconception, unaccompanied patients often ask the nurses to perform small personal tasks, making the nurses feel that they are not professionally valued. This cluster contains themes such as “being treated like an attendant,” “high expectations from caregivers and patients,” and “patients who do not hesitate to ask for personal favors.”

### Being treated like an attendant

As it is advertised by the government as a ward without the need for a guardian or caregiver, there was a perception that the integrated nursing care ward’s personnel would do everything for the patient. As participant 5 said, *“I think the perspective is that as the patients paid more to use integrated nursing care services, they can treat the nurse as a caregiver.”*

### High expectations from family and patients

Patients and guardians who used the integrated nursing care ward had heard about the service through hospital promotions. After acquiring information about the services and paying the additional costs prior to hospitalization, patients were admitted to the ward with considerably high expectations of the nurses. According to participant 2, *“People have high expectations, which lead them to expect servitude in our country. A nurse must become a servant and obey and fulfill all the requests.”*

Doctors working with them also lacked an understanding of the integrated nursing care ward, wishing for nurses to listen to all the needs of the patients and help them feel comfortable in the hospital. Additionally, if the nurses did not fully know the patient’s condition, they were rebuked.

*The doctors were critical of the nurses if they could not answer when asked about the patients’ conditions. They said, “This is the integrated nursing care ward. How do you guys not know?” Moreover, I think when the doctors want to do something for their patients, they ask the nurses to do it. Why the hell is that? Even the patient did not ask.* (Participant 12)

### Patients do not hesitate to ask for personal favors

In the integrated nursing care wards, there were no attendants nearby, and the patients had to do their own daily activities, which they perceived as being the nurses’ duty. Some patients did not ask about nursing treatments but requested clean underwear or socks from them or for them to go to a convenience store.

*They’re asking something too personal… I have heard of other places where they asked nurses to wash the patient’s underwear or something like that. It was a bit ridiculous when they asked me to wash their socks. Once, a patient said he could not hold a drink box because he had no strength, so he asked me to go to a convenience store and buy (smaller) drink boxes.* (Participant 14)

### Theme cluster 2: challenges owing to distorted perceptions of the integrated nursing care ward

Nurses working in integrated nursing care wards felt stressed when patients and families did not treat them appropriately. As some of the participants had doctoral or master’s degrees, such treatment made them question whether they should do such a job; they felt a sense of shame, disappointment, skepticism, deprivation, misery, and sadness and experienced confusion regarding their identity as a nurse.

### Being confused regarding roles and identity

While working in the integrated nursing care wards, the participants felt that their work as nurses was not merely to take care of patients. They were conflicted about whether their job was also to be a caregiver or assistant, and they experienced a loss of professional self-esteem. Participant 8 stated, *“I used to be proud of myself for being a nurse… Working in the nursing integration service ward, I felt… am I a nurse, an assistant, or a caregiver… Ha (sighs). My work feels trivial; I’m confused.”*

### Feeling the urge to resign

After watching colleagues resign because of the difficult work and new nurses leave without hesitation, the nurses of the integrated nursing care ward felt like quitting themselves. The work was physically difficult, and the stress from patients and caregivers who did not consider them nurses made them want to resign. Participant 11 stated, *“As the work is strenuous and it is very difficult to care for the patients, I keep thinking, ‘I want to resign.’ In fact, I even attended an interview to transfer to another ward.”*

### Experiencing physical symptoms of stress

Nurses experienced more stress in the integrated nursing service ward than in the general ward and suffered from insomnia, depression, shortness of breath, and indigestion. Participant 14 observed, “*In the early days of working in the integrated ward, I had insomnia. Others reported that they were depressed. Some people had trouble breathing. I have gray hair. I think stress makes people old (laughs).”*

### Theme cluster 3: loneliness and fighting alone

Nurses working in the integrated nursing service ward did not feel that they were cooperating with people in different occupations. Even when a patient suffered a fall, all the responsibility was placed on the nurse. The doctors who visited the ward also wanted the nurses to fulfill all the needs of the patients, assuming that that was the role of a ward nurse.

### Unable to leave the ward even for a moment due to the fear of patients falling

One of the most serious accidents that can occur in the integrated nursing care ward is a fall. As there are no attendants by their side, when patients go to the restroom or leave the room by themselves, they are at risk of falls. As a result, the nurses did not take sufficiently long lunch breaks.

*In general, guardians accompany patients, but here, there are none. When we go out to eat, we feel extremely anxious about the patients’ safety. Patients always fall when I am not there. After that, it becomes my fault. So, compared to other wards, we are anxious while eating. So, I rarely eat well.* (Participant 11)

### Responsibility rests solely with the nurse

When a problem arises with a patient, all responsibility is borne by the nurse. If the division of work is ambiguous, it is common to pass the responsibility onto the nurses as far as possible, with colleagues from other departments evading responsibility.

*I feel there’s nothing to protect me while I am taking care of patients, and it’s the nurse’s responsibility if anything happens, so the profession of a nurse often feels a bit miserable. Sometimes, the work of nurses and assistants becomes somewhat ambiguous. Then, the assistants try to avoid it and make us do it.* (Participant 1)

### Theme cluster 4: humiliation and harassment by patients

While working in the integrated nursing care ward, the nurses were ridiculed, which they had never experienced working in a general ward. This would not have been possible if there had been an attendant at the patient’s side. The patients said “thank you” in front of the nurses but narrated a different story to others behind their backs. When the patients tried to treat them like slaves or maids, the nurses felt ridiculed, which distressed them.

### Exposure to verbal abuse and sexual harassment

Despite the title of “nurse,” the patients called them by “hey,” “hello,” and even “yo, young lady.” Nurses in the general ward never changed a patient’s diaper or cleaned their buttocks; here, they were sexually harassed while helping the patients clean themselves, which the nurses attributed to working in the integrated care ward.

*At that time, I was changing a diaper, and the patient said, “Is this because you want to touch me?” Huh….hell… I felt really bad. Further, the patient was making physical contact by gently touching my waist. Ahhh… (takes a deep breath) I do not think this would have happened if the patient had an attendant.* (Participant 2)

### Patients’ different behaviors to their face and behind their back

The patients expressed their gratitude in front of the nurses but complained about them to others. The visiting families heard the patients’ complaints and in turn complained to the nurses.

*They encourage us to our face, but when the family comes, the patients tell them they have been neglected… I explained to the family, but they didn’t listen at all and shouted, “If you can’t allow us a personal caregiver, shouldn’t you do better?” At that time, I really wanted to quit.* (Participant 5)

### Being ridiculed

Nurses came across many patients who threw tantrums. On one occasion, a patient called and pretended to be in an extremely urgent situation, leaving the nurse bewildered.

*The patient pressed the call bell as if something really serious had happened. When I reached them, it was about a wet towel that had fallen… oh my god… I was really upset because I felt he was really making fun of me.* (Participant 4)

#### Theme cluster 5: practicing textbook holistic care

This theme cluster is about nurses being reminded of the meaning of nursing in an integrated care service ward. In general wards, caregivers or attenders perform some nursing tasks; however, in integrated nursing wards, nurses must cover the whole gamut for patients. Therefore, the nurses understood everything about the patient’s condition and felt they were caring for them attentively. Upon realizing that they were aware of the patient’s condition from head to toe, including small changes in skin condition, the nurses understood the meaning of holistic care, which they had previously only encountered in textbooks. Additionally, it was possible to identify the patient’s changing condition before an emergency arose, thereby preventing the transition to a critical condition.

### Assessing the patient from head to toe

This theme refers to the experience of providing holistic care that nurses are supposed to provide but previously could not because of the need to attend to many patients. The comprehensive assessment of patients is one of the most important tasks for a nurse by revealing the current condition of the patient and facilitating an adequate care plan. In general wards, the focus was on the patient’s special situation, mostly relying on the caregiver for information. In the integrated nursing services ward, nurses could observe the patient carefully and accurately assess the situation.

*I can observe everything from head to toe. As there is no caregiver, there are more opportunities to interact with the patient. When I work, I really enjoy it and take pride in the thought of being professional. I think that this is the merit of integrated nursing care services.* (Participant 14)

#### Caring for patients attentively and specifically

When caring for a patient in the integrated care service ward, the nurses felt that they knew everything about the patient in minute detail. In the general ward, they had little experience of directly seeing the patient’s urine volume or the color of the patient’s sputum.

*When nurses work in an integrated nursing service ward, I do not think they should just do what they do in a regular ward. They have to take a closer look, for example, when a patient urinates, the amount, color, etc. We do the suction by ourselves, so the smell, quantity, and color are observed. These are things that we asked the caregivers who were with the patients in the general ward. (Participant 9)*

#### Quickly detecting the patient’s condition

As there are no attenders in the integrated care services ward, nurses tend to visit the patient more often. If a patient was at a high risk of falling, they attended to their computer work in the vicinity of the patient. Additionally, the patient’s condition could be identified in advance before it worsened, preventing emergency situations.

*I am sure there are many rounds done in the integrated care service ward. It’s not just me but also my assistant, so if we alternate, it’s almost once every 30 min. That is why we could catch changes in the patient’s condition quickly and take action. For example, a diabetic patient was sitting on the bedside in a cold sweat and suddenly fell backward. I ran, caught him, and treated him immediately. I think I reacted quickly.* (Participant 4)

#### Theme clusters 6: the satisfaction felt only in the integrated nursing care ward

This theme cluster refers to the good experiences unique to an integrated service ward. Notably, the satisfaction of patients using the integrated nursing care ward is high, and family members who cannot afford to take care of them because of work or school often express their gratitude. The nurses considered this appropriate to the original purpose of the integrated nursing service. As there was no attendant around the patient, they trusted their nurse completely and actively cooperated with treatment. The nurses took pride in providing care because it seemed to convey to their patients that they were professionals.

### High patient satisfaction

Patients admitted to the integrated service ward were satisfied with the service. They were extremely satisfied with the environmental aspect—a quiet and calm atmosphere—and they were content with the meticulous care of the nurses.

*I definitely think that the satisfaction level is higher than in the general ward. The general ward is crowded like a flea market because caregivers are on duty 24 h a day. Many complaints arise due to the caregivers, not the patients. The ward here is quiet, so it’s good for the patients.* (Participant 9)

### Gratitude for reducing the burden on the family

The caregivers who understood the original purpose of the integrated nursing care ward were grateful. In modern society, as most families are dual-income, they cannot take care of the patients themselves. These families express their gratitude to the nurses every time they visit the patient.

*Everyone is busy with work these days, and if one of the family members becomes sick, someone has to make a sacrifice. Those who work have to take leave or hire a caregiver, but in the integrated nursing care ward, the burden on the family is significantly reduced. That is why many caregivers visit to say thank you.* (Participant 14).

## Building trust

In the integrated service ward, patients trust the nurse more owing to the enhanced contact. Nurses said that frequent rotations allowed them to communicate and give patients confidence that they were getting the care they needed.

*In the general ward, there are many patients, so it is difficult to understand if the patients are sleeping well at night. However, as I often go on rounds here, if they cannot sleep, I can help them. I think trust builds because I take care of them directly.* (Participant 6)

## Discussion

This study explored the experiences of nurses working in integrated service wards using Colaizzi’s [20] phenomenological research method. In-depth interviews were conducted with 17 nurses having more than one year of working experience in an integrated service ward. The data analysis yielded six thematic clusters: “distorted perceptions of the integrated nursing care ward,” “challenges owing to the distorted perceptions of the integrated nursing care ward,” “loneliness and fighting alone,” “being ridiculed,” “practicing textbook holistic care,” and “the pride felt only in the integrated nursing care ward.” Our findings show that the nurses working in the integrated service ward experienced stress owing to distortions and misunderstandings regarding their role, contrary to those in the general ward, but found significance in providing holistic care.

When the integrated nursing service ward was first introduced in Korea, the government emphasized the advantage of being able to receive care from a professional nurse under the slogan of a “ward without a caregiver” [21], which shaped the public perception of the nurses of the integrated ward as performing the role of caregiver, which the nurses working in these wards call a grave distortion. Although the cost of the integrated nursing service varies between hospitals, it is evident that the patient bears a greater monetary cost than in a general ward [21]. Thus, the patient and their family seek greater benefits and expect that nurses will take care of the patient with focused attention, assuming them to be attendants available for personal matters. Other studies [22, 23] reporting that patients treat nurses as personal assistants are consistent with the results of this study. 777However, in Korea, the patient-nurse ratio in the integrated nursing service ward ranges from 7:1 to 10:1. Under these circumstances, it is difficult for nurses to act as bedside caregivers for all patients.

Such distortions are a major stressor for nurses, leading them to experience skepticism or frustration regarding their identity. Although nurses are professionals who do their best for the health and well-being of their patients, they experience disrespect. Such stress causes physical symptoms, most commonly insomnia, depression, shortness of breath, and indigestion. As many studies have linked stress to serious illness, it should be avoided [24, 25], and when stress is unavoidable, it is important to avoid constant exposure and practice stress management [26]. However, nurses in the integrated service ward are continuously exposed to stress and there are no institutional protocols for stress management. Therefore, future research must focus on how to reduce nurses’ occupational stress and manage it more effectively by understanding and addressing their needs. Moreover, many nurses working in the integrated ward desired to leave when they saw their peers leave or change jobs. Research shows that nurse turnover occurs most often when professionalism is not recognized [18]. However, even in such a situation, if appropriate compensation is provided, the turnover rate can be lowered and job satisfaction increased [19]. Therefore, it is necessary to develop resources to lower the turnover intention of nurses in the integrated service ward by improving the compensation system accordingly.

Nurses working in the integrated service ward undertake considerable responsibility. Although there are nursing assistants and other staff, all the responsibility for patients’ problems falls on the nurse’s shoulders. Falls are considered the most serious problem in the integrated service ward, and when a patient falls, it is assumed that the nurse was not attentive enough [27]. For example, if a patient falls during a nurse’s mealtime, the responsibility still lies with the nurse; therefore, they cannot take a proper break. Because the division of duties in an integrated nursing ward is still unclear, physicians often require nurses to do everything that a patient wants because they themselves do not fully understand the scope of the services [28, 29]. There is a need to clearly delegate work across the inter-disciplinary team based on skills and assign responsibilities appropriately.

In the integrated nursing care ward, there is also a problem of harassment due to the absence of an attendant. The patient may treat nurses differently if there is no family at their bedside. The most serious problem the nurses experience is being directly attacked, as through sexual harassment or abusive language.

Moreover, patients may try to order the nurses around using the excuse that there are no family members by their bedside; when a family member visits, the nurse experiences completely different patient behavior. Conversely, there were complaints of patients expressing kindness and gratitude to the nurses when they were along but deprecating them when family members visited. These experiences made the nurses feel ridiculed, which they felt could have been avoided with the presence of a caregiver [30]. Therefore, it is necessary to assiduously provide patients with correct information and educate them on how to use the integrated ward while respecting the staff.

One of the cherished experiences of nurses working in the integrated service ward is the provision of holistic nursing, as described in textbooks. Usually, in a general ward, a problem-focused assessment of the patient is performed with a guardian or caregiver always at the patient’s side to provide information. In the integrated service ward, nurses can assess the patient from head to toe, making it possible to better understand the patients’ circumstances and check the condition of the patient’s skin and the color and amount of their bodily excretions.

As a result, an advantage of the integrated nursing care ward is that in situations where the patient’s condition changes rapidly, it is possible for nurses to recognize the changes quickly and provide treatment before the patient deteriorates [31].

According to the patients who have used the integrated service ward, frequent contact with the nurses helped form rapport and build trust easily, and the patients’ satisfaction levels were high. Families can also maintain their daily routine by using the integrated service ward, which has the special feature of reducing the burden of patient care while providing a high level of satisfaction [29]. Thus, while nurses working in the integrated service ward experienced stress from distorted perceptions of their role, they could practice holistic nursing and recall the traditional view of the profession. Currently, integrated nursing care services in Korea have not been clearly defined; thus, it is fundamental to improve awareness of the entire treating team and update the compensation system for nurses accordingly. These findings can stimulate the provision of practical assistance to nurses working in integrated care wards, contributing to nursing practice. Additionally, this study can serve as a foundation for subsequent research aimed at devising measures to improve nurses’ working conditions.

## Conclusion

This study provides insights into nursing practice and education. We utilized an inductive, phenomenological approach to delve into the practical experiences of nurses in integrated nursing care wards. The first theme cluster, “distorted perceptions of the integrated nursing care ward,” explores nurses’ challenges stemming from misleading government communication and inadequate public information. The second theme, “challenges owing to the distorted perceptions of the integrated nursing care ward,” examines the stressors and their effects on nurses’ self-esteem and physical well-being. “Loneliness and fighting alone” highlights nurses’ isolation in the ward despite working with colleagues from various other disciplines. “Being ridiculed” concerns nurses’ feelings about their unfair treatment by patients. The fifth theme, “practicing textbook holistic care,” describes the opportunity to adopt a unique approach to patient care. Finally, “the satisfaction felt only in the integrated nursing care ward” reflects nurses’ job satisfaction and trust-building with patients.

This study’s focus on nurses’ distinctive experiences in integrated care wards offers insights to enhance operational systems, develop policies to improve working conditions, and establish appropriate procedures. Future research could quantify nurses’ experiences, assess their work satisfaction, and address the physical and mental challenges they encounter in integrated care settings.

## Data Availability

As per the rules of our Research Ethics Review committee, participants were assured that their interview transcripts would not be made public. However, the corresponding author can make anonymized data available on reasonable request.

## Abbreviations

NHIS: National Health Insurance Service
